# Synchronization dependent on spatial structures of a mesoscopic whole-brain network

**DOI:** 10.1371/journal.pcbi.1006978

**Published:** 2019-04-23

**Authors:** Hannah Choi, Stefan Mihalas

**Affiliations:** 1 Department of Applied Mathematics, University of Washington, Seattle, WA, USA; 2 Allen Institute for Brain Science, Seattle, WA, USA; Institut de Neurosciences des Systèmes, FRANCE

## Abstract

Complex structural connectivity of the mammalian brain is believed to underlie the versatility of neural computations. Many previous studies have investigated properties of small subsystems or coarse connectivity among large brain regions that are often binarized and lack spatial information. Yet little is known about spatial embedding of the detailed whole-brain connectivity and its functional implications. We focus on closing this gap by analyzing how spatially-constrained neural connectivity shapes synchronization of the brain dynamics based on a system of coupled phase oscillators on a mammalian whole-brain network at the mesoscopic level. This was made possible by the recent development of the Allen Mouse Brain Connectivity Atlas constructed from viral tracing experiments together with a new mapping algorithm. We investigated whether the network can be compactly represented based on the spatial dependence of the network topology. We found that the connectivity has a significant spatial dependence, with spatially close brain regions strongly connected and distal regions weakly connected, following a power law. However, there are a number of residuals above the power-law fit, indicating connections between brain regions that are stronger than predicted by the power-law relationship. By measuring the sensitivity of the network order parameter, we show how these strong connections dispersed across multiple spatial scales of the network promote rapid transitions between partial synchronization and more global synchronization as the global coupling coefficient changes. We further demonstrate the significance of the locations of the residual connections, suggesting a possible link between the network complexity and the brain’s exceptional ability to swiftly switch computational states depending on stimulus and behavioral context.

## Author summary

In a previous study, a data-driven large-scale model of mouse brain connectivity was constructed. This mouse brain connectivity model is estimated by a simplified model which only takes in account anatomy and distance dependence of connection strength which is best fit by a power law. The distance dependence model captures the connection strengths of the mouse whole-brain network well. But can it capture the dynamics? In this study, we show that a small number of connections which are missed by the simple spatial model lead to significant differences in dynamics. The presence of a small number of strong connections over longer distances increases sensitivity of synchronization to perturbations. Unlike the data-driven network, the network without these long-range connections, as well as the network in which these long range connections are shuffled, lose global synchronization while maintaining localized synchrony, underlining the significance of the exact topology of these distal connections in the data-driven brain network.

## Introduction

Structural neural connectivity and its implications for brain function have been a long-sought subject in neuroscience. Many previous studies have been limited either to small networks of few cells or coarser connectivity among larger brain regions [1–9], often binarized and without spatial information. Recent development of the Allen Mouse Brain Connectivity Atlas from anterograde fluorescent viral tracing experiments [10] provides us the unique opportunity to investigate precise weighted anatomical connectivity of the mammalian whole brain network. Combining the mesoscopic connectivity data with spatial information of the network, we seek a parsimonious representation of the weighted whole-brain network that captures salient network properties. Specifically, we investigate whether the network can be compactly represented solely based on the spatial dependence of the network topology.

Biological networks are inherently spatially constrained. Recent studies have shown that geographic constraints play a critical role in generating graph properties of real-world neuronal networks [5, 11–20], which cannot be fully captured by classical generative network models such as the small-world network [2] and the scale-free network [21]. Yet many of the studies are limited to binarized networks [11, 12, 17, 19, 20] and are focused explicitly on comparing graph theoretical measures [11, 13–20]. In this paper, we examine spatial embedding of a weighted whole-brain connectivity, and ask whether spatial dependence alone can depict the full computational capability of the brain network by studying dynamics of the network.

By analyzing the latest connectivity data from a new mapping algorithm, we find that the network connectivity strongly depends on its spatial embedding, with spatially close brain regions strongly connected and distal regions weakly connected. We study the precise relationship between connectivity and distance, and investigate possible computational roles of positive residual connection strengths that are not captured by the spatial dependence. To probe the possible implications of the residual connections on the network dynamics, we construct a network of phase oscillators with the data-driven adjacency matrix and compare its dynamics to those of the oscillator network with the connections strictly dependent on distance. We analyze spatial structures of synchronization by measuring the order parameter for varied amounts of global coupling coefficient. We further examine the strong connections between distal brain regions by studying network dynamics when fractions of the strong residual connections are added to the spatially constrained network. Finally, we relocate the positive residuals either to connections between nearby brain regions or to different fractions of longest-range connections, thus increasing the connection strengths for the spatially close or distal brain regions while eliminating sparse, strong connections spread across different edge lengths. The networks restructured this way maintain overall connection strength of the brain network but have a connectivity topology different from that of the brain network. By comparing dynamics of such restructured networks and the data-driven whole brain network, we show that the spatial locations of the strong positive residuals are important. Specifically, our study reveals that strong connections distributed over the brain network across many length-scales enhance the capability of the system to switch between asynchronous and synchronous states, underlining the significance of the existence of these connections. The network without these long-range connections, as well as the network in which these long-range connections are shuffled, when pushed by perturbations or low coupling coefficient, lose global synchronization but maintain local synchronization over small spatial scales. In the same conditions, the data-driven network loses synchronization over all spatial scales. It is interesting to speculate that this phenomenon is necessary for the integrative processes necessary for global cognitive functions.

## Results

### Spatial dependence of the mouse whole-brain connectivity

**Fig 1:**
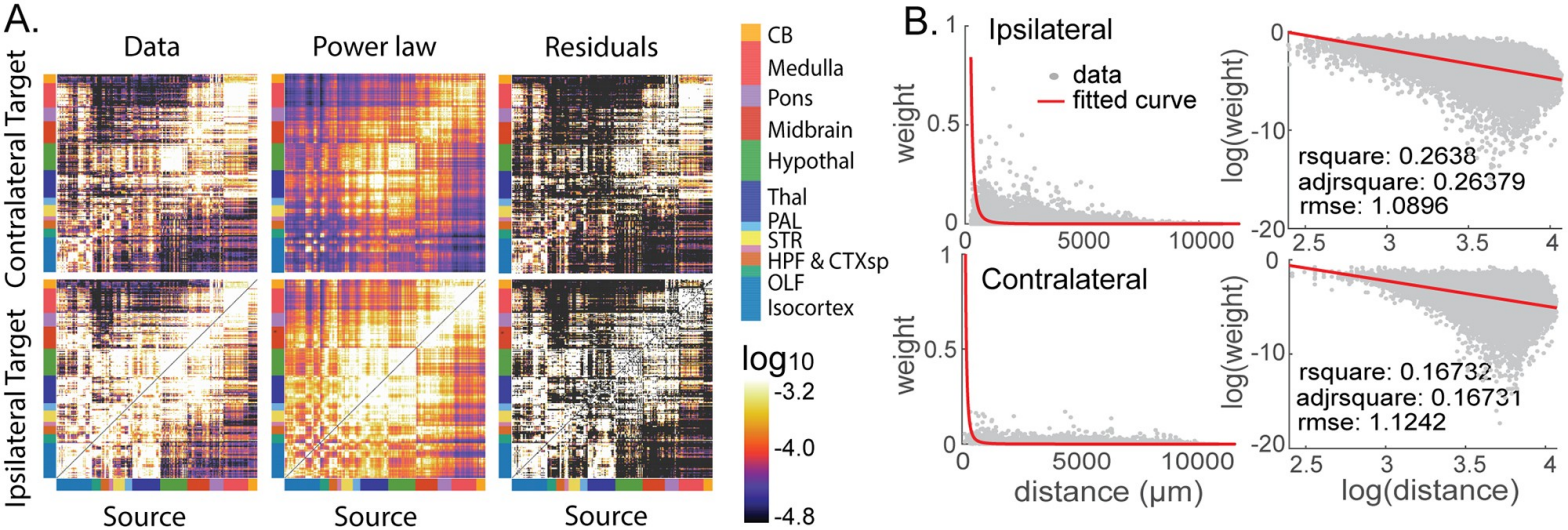
Connectivity matrices constructed from viral injection data and the power-law dependence on distance. (A) Connectivity matrix from viral tracing data (left); reconstructed connectivity from the power-law dependence on distance between nodes (middle); residual connection strengths of the data-driven network above the power-law distance dependence. We show 244 brain regions divided in to coarser major brain divisions defined in the Allen Mouse Brain Reference Atlas. These divisions are: Isocortex, Olfactory Bulb, Hippocampus, Cortical Subplate, Striatum, Pallidum, Thalamus, Hypothalamus, Midbrain, Pons, Medulla, and Cerebellum. (B) Connection strengths as a function of distance between brain regions (left panels). The connections obtained from experiments (gray) are fit by a power law (red) on the log scale with base 10 (right panels). Inset: Goodness of fit.

The mesoscopic mouse whole-brain connectivity was constructed based on viral tracing experiments available on the Allen Mouse Brain Connectivity Atlas [10] with a recently developed interpolative mapping algorithm [22]. This produced a weighted and directed structural connectivity matrix with 244 brain regions as source nodes and 488 brain regions as ipsilateral (244) and contralateral (244) target nodes. By combining the ipsilateral and contralateral connections for each hemisphere, we constructed a whole-brain connectivity matrix with 488 nodes. The data-driven mouse brain network is shown in Fig 1A, left column.

We analyzed the relationship between connection strength and spatial distance between brain regions in the data set. In accordance with previous studies on brain networks [5, 15–20], the connectome strongly depends on the spatial embedding; connections are stronger between spatially close regions and weaker between distal regions. Specifically, the connection strengths decrease with distances between brain regions following a power law (Fig 1B) rather than an exponential relationship, in agreement with previous studies on Allen Mouse Brain Connectivity data [18, 22]. Additional details on the fitting are available in Methods (“Dependence of connection strengths on interregional distance”).

We constructed adjacency matrices for the ipsilateral and the contralateral networks based on the power-law relationship, as shown in Fig 1A, middle column. While the general trend of decrease in connection strength with distance is clear and well-predicted by a power law, there are also a number of residual connection strengths that are not captured by the power-law relationship (Fig 1A, right column).

**Fig 2:**
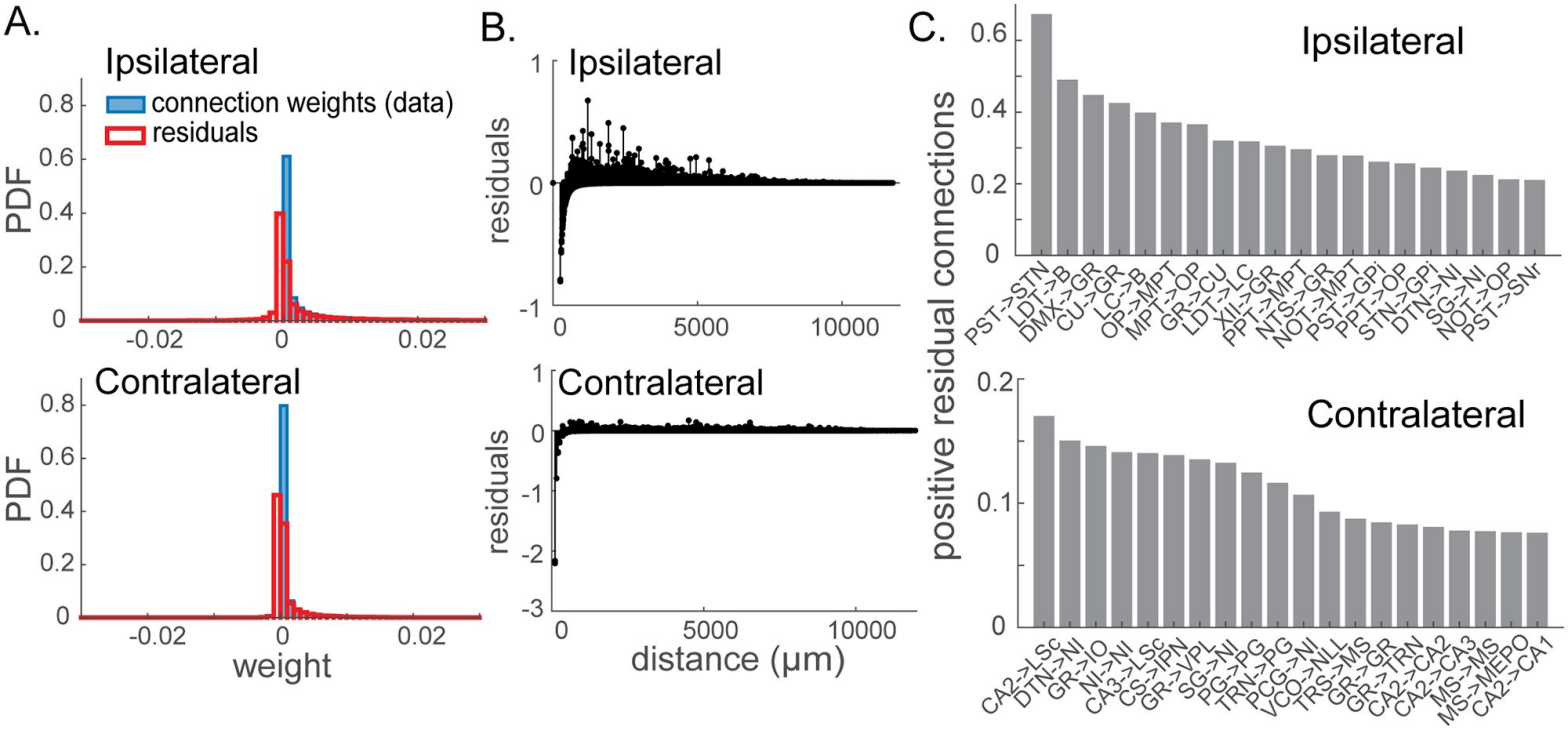
Residual connection weights unexplained by the power-law distance dependence. (A) Distributions of the connection strengths from the data (blue) and the residual connection strengths (red). The x-axes are restricted here to better visualize the positive tails and many of the residuals clustered around zero. (B) Residual connection weights as a function of distance between nodes. (C) Directed pairs of brain regions with large positive residual connections. These represent pairs of regions with connections stronger than predicted by the interregional distance. For reference on the acronyms of the regions, see the Allen Mouse Brain Reference Atlas (https://mouse.brain-map.org/static/atlas).

To understand the structure and effects of the residual connection weights that are not captured by the power-law dependence on distance, we had a closer look at these residuals. For both ipsilateral and contralateral connections, a long, positive tail is observed in the distribution of residual connection weights, suggesting strong distal connections above the power-law dependence on distance (Fig 2A and 2B). The strongest 20 residual connections are plotted in Fig 2C. We observed that for the ipsilateral network, connections from preparasubthalamic nucleus (PST) to subthalamic nucleus (STN), laterodorsal tegmental nucleus (LDT) to Barrington’s nucleus (B), dorsal motor nucleus of the vagus nerve (DMX) to gracile nucleus (GR), cuneate nucleus(CU) to gracile nucleus (GR), and locus ceruleus (LC) to Barrington’s nucleus (B) are a few examples of the strong distal connections unexplained by the power-law dependence on distance. For the contralateral connectivity, on the other hand, many of the strongest residuals above the power-law relationship include connections between the same regions in different hemispheres as well as connections to and from hippocampal areas.

### Phase oscillators and network coherence measures

Do these positive residual connections between distal regions have any computational significance? In other words, can we capture the full computational capacity of the mesoscopic brain network with connectivity governed by strictly distance-dependent rules, with the residuals removed? To test this, we compare dynamics of the data-driven brain network to those of an artificial, strictly distance-dependent network generated by the power-law relationship. Specifically, we built a network of coupled phase oscillators whose coupling strengths are described by the weighted adjacency matrix of the data-driven brain network or the power-law distance-dependent connectivity. Each of these Kuramoto-type phase oscillators corresponds to a brain area. Kuramoto-type coupled phase oscillators have been widely used to model oscillatory brain dynamics [23–25]. The phase of region *i*, represented by *θ*_*i*_, is described by:

$$\begin{array}{c} \begin{array}{c} {\dot{\theta }}_{i}=\omega_{i}+k\sum_{j=1}^{N}A_{ij}\sin\left(\theta_{j}\left(t-\tau_{ij}\right)-\theta_{i}\left(t\right)\right)+\eta_{i}\left(t\right) \end{array} \end{array}$$ (1)

where *ω*_*i*_ denotes the natural frequency, and *k* describes the coupling coefficient. *A*_*ij*_ is the adjacency matrix of the network. For the case of the data-driven brain network, *A*_*ij*_ = *J*_*ij*_ where *J*_*ij*_ indicates the adjacency matrix obtained from viral tracing data, for both ipsilateral and contralateral connections. For simulations of the artificial, distance-dependent network, *A*_*ij*_ = *K*_*ij*_ indicates the adjacency matrix constructed by making the connection weights strictly follow the power-law dependence on distance. The last term *η*_*i*_(*t*) represents an additive Gaussian white noise with zero mean (〈*η*_*i*_(*t*)〉_*t*_ = 0) and variance $\sigma_{n}^{2}/T({\langle \eta_{i}\left(t\right)\eta_{j}\left(t^{\prime }\right)\rangle }_{t}=\delta_{ij}\delta \left(t-t^{\prime }\right)\sigma_{n}^{2}/T)$, where *δ*_*ij*_ is the Kronecker delta and *δ*(⋅) denotes the Dirac delta function. The standard deviation *σ*_*n*_ is in radians and *T* is a timescale, which is set to 1 second in our study. *N* denotes the number of nodes of the network, which is 488 in our whole-brain simulations. The natural frequencies *ω*_*i*_ are randomly chosen from a symmetric, unimodal distribution *g*(*ω*). In this paper, we used a Gaussian distribution with the mean at 40 Hz and the standard deviation *σ*_*d*_ for *g*(*ω*), as done in other studies of modeling large-scale brain dynamics with phase oscillators [24–27]. Note that this falls within a frequency range of gamma rhythms (30-80 Hz) that are frequently observed in oscillatory brain dynamics.

Numerous previous studies have shown the importance of distance-dependent delays in networks of oscillators [28–33]. For example, time delays can destabilize synchrony in neuronal networks, leading to travelling waves [29–33]. To reproduce synaptic and axonal conduction delays dependent on connection distance, we incorporated distance-dependent time delays in our model as done in other studies [23–27, 34–37]. In the rodent brain, the conduction velocity ranges from values as low as 0.5 (m/s) to much higher speed around 10 (m/s) depending on various factors such as axonal myelination [32, 38, 39]. Experimental studies show that the propagation speed distributions peak in between 2-5 (m/s) [32, 39]. While time delays are heterogeneous over different regions in the brain, we simplified the model by using a fixed conduction speed at 3.5 (m/s) for the whole brain, which falls in the middle of the propagation speed distribution peak. In Eq 1, the distance-dependent time delay between areas *i* and *j* is denoted by *τ*_*ij*_, which is computed by dividing the Euclidean distance *d*_*ij*_ between nodes *i* and *j* by the fixed conduction speed.

We investigated the dynamics of the data-driven network and the power-law generated network using Eq 1, and measured the network coherence by calculating the “universal” order parameter *r*, recently proposed in [40] as following:

$$\begin{array}{ll} r & \equiv \frac{1}{\sum_{i=1}^{N}k_{i}}\sum_{i,j=1}^{N}A_{ij}\langle Re\left(e^{\text{i}\left(\theta_{i}-\theta_{j}\right)}\right)\rangle_{t}\\ & =\frac{1}{\sum_{i=1}^{N}k_{i}}\sum_{i,j=1}^{N}A_{ij}{\langle \text{cos}\left(\theta_{i}-\theta_{j}\right)\rangle }_{t} \end{array}$$ (2)

where $k_{i}=\sum_{j=1}^{N}A_{ij}$ is the input strength of node *i*. Unlike the original order parameter which was proposed by Kuramoto [41, 42] for all-to-all coupled phase oscillators (see Eq 6 in Methods), the universal order parameter [40] was developed to quantify coherence in more general, weighted networks of oscillators. The universal order parameter accounts for the network topology and its influence on the phase coherence. Therefore, we can compare network coherence in topologically different weighted networks even when their total connections strengths are not the same. Furthermore, the universal order parameter captures partially phase-locked states accurately. To quantify different degrees of network coherence and to visualize localized and global synchrony, we measured the universal order parameter, both for the whole network of oscillators (Eq 7 in Methods) as well as for subnetworks of different spatial scales (Eq 8 in Methods). To compute order parameters of subnetworks on the spatial scale *d*, we measured the averaged phase difference for each node *i*, with all the other nodes that are within the given spatial distance *d* from the node *i*. Thus computed averaged phase difference for each node is weighted by the inverse of input strengths to the given node *i* provided by its neighbors within the distance *d* from the node, and summed over all regions in the whole network. By thus computing the order parameter for the subnetworks, we describe the order parameter as a function of distance.

Obtaining an explicit, analytical relationship between the order parameter and generalized network structures has been a challenging problem in studies of phase oscillators on complex networks [43, 44]. While analytical expressions for the order parameter as a function of the adjacency matrix have been derived in previous works, these mean-field approaches are based on strong assumptions of a large network with sufficiently high average degree, valid only near the onset of synchronization [41, 45–48]. Existing analytical approaches, therefore, are not applicable to the complex mesoscopic brain network of a finite size. We thus address the relationship between the network coherence and the network structure by computing the order parameter based on numerically obtained time series of the oscillators.

**Fig 3:**
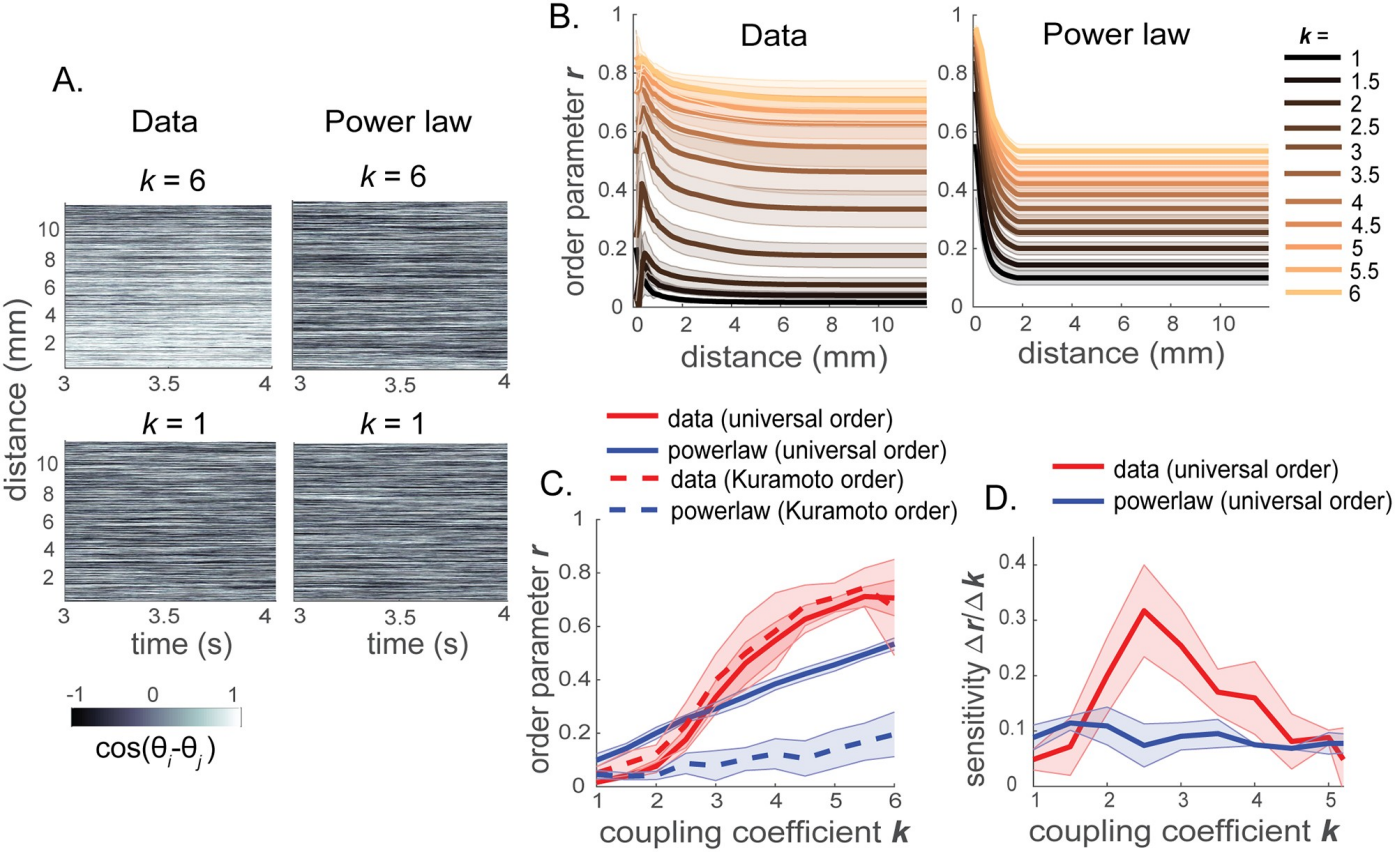
Local and global synchronization of the data-driven brain network and the power-law network. (A) Phase differences cos(*θ*_*i*_ − *θ*_*j*_) of pairs of nodes (*i*,*j*) as a function of time (x-axis) and distance between nodes (y-axis) for the data-driven and power-law networks. For both networks, the intrinsic frequencies of the oscillators were at 40 Hz (*σ*_*d*_ = 0) and the standard deviation of the Gaussian white noise was fixed at *σ*_*n*_ = 2. (B) Universal order parameter *r* for subnetworks at different spatial scales, for the data-driven and the artificial power-law networks with different amounts of global coupling coefficient *k*. Order parameter *r* is averaged over 10 simulations. (C) Universal order parameter (solid) and Kuramoto’s original order parameter (dotted) for the whole networks of the data-driven connectivity (red) and the power-law distance-dependent connectivity (blue), as a function of global coupling coefficient *k*. (D) Sensitivity of the order parameter as a function of the coupling coefficient *k*, for the data-driven connectivity (red) and the power-law approximated connectivity (blue). Lines and shades correspond to the mean and the standard deviation over multiple simulations.

Phases were initialized randomly, and Eq 1 was integrated numerically using the Forward Euler method, with a sufficiently small time step Δ*t* = 10^−4^ (s) for 4 seconds (*N*_*t*_ = 40000 steps), until a stationary state is reached. In our simulations, the time step size Δ*t* = 10^−4^ (s) satisfies the condition $\Delta t\leq 0.01/\max(\max\left(k\cdot A_{ij}\right),0.05\mu ,\frac{\sigma_{n}^{2}}{2T},1)$, as in [37]. The data from the first *N*_*t*_/2 steps are discarded in measuring the order parameter. The order parameter, representing network coherence, can be modulated by the global coupling coefficient *k*, the standard deviation *σ*_*d*_ of the intrinsic frequency distribution, and the standard deviation *σ*_*n*_ in the additive Gaussian white noise. In this paper, we computed the order parameter using Eq 8 in Methods for varied global coupling coefficient *k*, with the standard deviation of the natural frequency distribution fixed at *σ*_*d*_ = 0 (Hz) and the standard deviation of the Gaussian noise fixed at *σ*_*n*_ = 2 (rad). For each value of coupling coefficient *k*, we performed 10 independent runs, and plotted the average and the standard deviation of the order parameter as a function of distance between nodes (Fig 3B) as well as a function of global coupling coefficient *k* (Fig 3C). We also show the order parameter as a function of the coupling coefficient *k*, with a nonzero standard deviation in the natural frequency distribution in the Supporting Information S2(C) Fig. In this figure, the order parameter was averaged over 100 repeats with *σ*_*d*_ = 0.2 (Hz) and *σ*_*n*_ = 2 (rad), to offset different effects of each configuataion of the intrinsic frequencies due to the nonzero *σ*_*d*_.

When the standard deviation of the white noise is held constant, increasing the coupling coefficient *k* with fixed *σ*_*d*_ has qualitatively the same effect as decreasing *σ*_*d*_ with *k* fixed, as the ratio of *k*/*σ*_*d*_ determines the network coherence. The same is true for decreasing the amount of *σ*_*n*_. We show that varying *σ*_*n*_ and *σ*_*d*_ produces the same qualitative results as with varying *k* in the Supporting Information S2 Fig. When *σ*_*n*_ is varied, the intrinsic frequency distribution and the coupling coefficient are held constant, at *σ*_*d*_ = 0 and *k* = 3, and the order parameter was averaged over 10 repeats. When *σ*_*d*_ is varied, the other two parameters are fixed at *σ*_*n*_ = 0 and *k* = 2, and the order parameter was averaged over 100 repeats to account for the dependence of the time series on different configurations of the intrinsic frequencies. By computing the sensitivity of the network synchronizability on perturbation in each of these parameters—*k*, *σ*_*n*_, or *σ*_*d*_, we show that the observed trend in the data-driven brain network and the power-law approximated network is robust.

### Sensitivity of the network coherence

In Fig 3A, we show the phase difference cos(*θ*_*i*_ − *θ*_*j*_) for pairs of nodes (*i*,*j*) plotted against time and distance between the nodes. Interestingly, for the same amount of change in coupling coefficient Δ*k*, the data-driven brain network switches between an asynchronous state and near-global synchrony, while the power-law governed network fails to make such a drastic change in synchronization state. This difference is manifested in the order parameter. Fig 3B shows the universal order parameter (Eq 8) for subnetworks of different spatial ranges. When *k* is small, both the data-driven brain network and the power-law approximated network have overall low order parameters. In both cases, however, the order parameter is higher for small spatial scales, indicating that there is some spatially localized coherence in the networks due to the general trend of decreasing connection strength with distance between the connected regions.

At a finer scale, we also observe a small amount of initial increase in the order parameter for the shortest-range connections (110-346 *μm*) followed by a slow decrease in the order parameter as a function of distance in the data-driven brain connectome. Such an initial increase in the order parameter is not seen in the power-law estimated network. However, this initial rise at the very small length-scale should not be over-interpreted, because the experimental data are based on the mesoscopic measurements which are not accurate for distances less than 300-500 *μm*. In the viral tracing experimental data, the average distance to the closest injection is typically 500 *μm* at source level, which limits resolution [10, 22].

In the data-driven brain network, increasing the coupling coefficient *k* results in a transition from partial coherence to near-global synchrony, manifested by increased order parameters across a range of spatial scales (Fig 3B, left column, Data). However, in the artificially generated, strictly distance-dependent network, the same amount of change in global coupling coefficient does not induce such a leap in the network coherence state as in the real brain network (Fig 3B, right column, Power law).

Such trends can be also visualized in the order parameter for the whole network. The overall universal order parameter increases with global coupling coefficient in both the data-driven and the power-law networks (Fig 3C). However, the change in order parameter is significantly larger in the data-driven brain network. This trend appears in both the single hemisphere network with only ipsilateral connections (Supporting Information S1 Fig) and the whole brain network with both ipsilateral and contralateral connections (Fig 3C). For comparison, Kuramoto’s original order parameter (Fig 3C, dotted) is also plotted. Because the original Kuramoto’s order parameter does not account for different connection strengths among different pairs of nodes nor measure coherence scaled to the overall degree of the network, we see that the Kuramoto order parameter is lower than the universal order parameter for the power-law network. Nevertheless, for either type of the order parameter, we observe that the data-driven brain network spans a larger range of coherence states than the power-law governed network.

These trends are more clearly portrayed by plotting the sensitivity of the order parameter (Δ*r*/Δ*k*) as the coupling coefficient *k* is varied (Fig 3D). We observe that the sensitivity remains relatively constant throughout the range of the coupling coefficient in the power-law approximated network. However, the sensitivity of synchronizability is considerably more variable in the data-driven network, peaking around *k* = 2.5. As the coupling coefficient increases, the sensitivity in synchronizability thus increases and then drops after reaching the maximum in the data-driven network, while the power-law approximated network is marked by relatively invariant, low sensitivity of the order parameter.

This result on order parameter can be manifested by a couple of simple measures we use here. To compare the maximum sensitivity of the order parameter to changes in the global coupling coefficient *k* (or any other parameters that modulate synchronizability, such as *σ*_*n*_ and *σ*_*d*_), we introduce a measure of the maximum sensitivity of synchronizability:

$$\begin{array}{c} \Gamma_{k}=\max_{k,k+\Delta k}(\frac{\Delta r}{\Delta k}). \end{array}$$ (3)

For the power-law network, the averaged maximum sensitivity of the order parameter is Γ_*k*_ = 0.1144 ± 0.0214. The maximum sensitivity of the order parameter is higher in the data-driven mouse brain network, at Γ_*k*_ = 0.3172 ± 0.0829. The higher value of the sensitivity measure Γ_*k*_ for the data-driven brain network indicates that a small amount of change in the coupling coefficient can induce a significant change in the network’s coherence state, in particular, within the range of *k* where Δ*r*/Δ*k* is maximum.

To evaluate spatial dependence of the order parameter, we use another measure that quantifies the difference between the order parameter for short-range subnetworks and the order parameter for the whole network. This measure is defined as:

$$\Gamma_{d}={\langle r\left(d=d_{\text{shortest}}\right)-r\left(d=d_{\text{longest}}\right)\rangle }_{k},$$ (4)

where *d*_shortest_ is the distance less than 570 (*μm*) that generates the highest order parameter value *r*(*d*), and *d*_longest_ is 11955 (*μm*) which is the longest connection length in the mouse whole-brain network. 〈⋅〉_*k*_ denotes averaging across varied coupling coefficient *k*, and *r*(*d*) is computed as defined in Eq 8. For the data-driven brain network and the power-law-driven network, Γ_*d*_ = 0.1851 ± 0.0706 and Γ_*d*_ = 0.5383 ± 0.0234, respectively. The larger Γ_*d*_ of the power-law network depicts a larger drop in coherence as the region of interest expands from the spatial vicinity to the whole network in the power-law network. In other words, the power-law network exhibits more localized coherence throughout a range of varied coupling coefficients.

We also confirmed that such difference between the data-driven brain network and the strictly distance-dependent, power-law network remains unchanged when the natural frequencies of the nodes are moved to 8 Hz and 20 Hz, which are in the ranges of theta (6-12 Hz) and beta (10-30 Hz) oscillations, respectively. Like gamma oscillations, theta and beta oscillations are frequently observed in the large-scale brain dynamics. While gamma oscillations are thought to be linked to cognitive processing and sensing, theta rhythms are observed in hippocampal LFP and thus believed to underlie memory formation. On the other hand, beta rhythms have been associated with movement preparation and motor coordination [23, 49, 50]. As with the natural frequencies at 40 Hz, simulations with intrinsic frequencies at 20 Hz and 8 Hz also predict that the synchronizability is more sensitive to changes in global coupling coefficient in the data-driven brain network than in the power-law approximated network (Supporting Information S3 Fig). With the realistic propagation speed 3.5 (m/s) and the longest connection at 11955 (*μm*) in the mouse whole-brain, the time delays in our model are quite small, and thus different intrinsic frequencies within the biologically realistic range induce qualitatively the same trend in synchronizability. It has been shown in previous studies that when the time delays multiplied by the intrinsic frequencies are sufficiently small compared to the coupling strengths in phase oscillators, the delay enters as a simple phase-lag [51, 52].

Our results indicate that in the real brain network, a small change in the global coupling coefficient induces a rapid transition between partial network synchrony and a more globally synchronized state, while in the network with connections strictly following a power-law dependence on distance, such a rapid transition to synchronization is not observed. We get qualitatively the same results when we vary parameters other than the coupling coefficient *k*, namely, *σ*_*d*_ and *σ*_*n*_, to modulate the network synchronizability. The order parameter is more sensitive to changes in the dispersion of intrinsic frequencies (*σ*_*d*_) and the standard deviation in the additive white noise (*σ*_*n*_) in the data-driven brain network than in the power-law governed network as well (Supporting Information S2 Fig). Therefore, the residual connection strengths that are not explained by the simple spatial rule may have some computational significance, enabling even small perturbations in cognitive or behavioral states to induce a transition to synchronization.

### Effects of strong long-range connections

**Fig 4:**
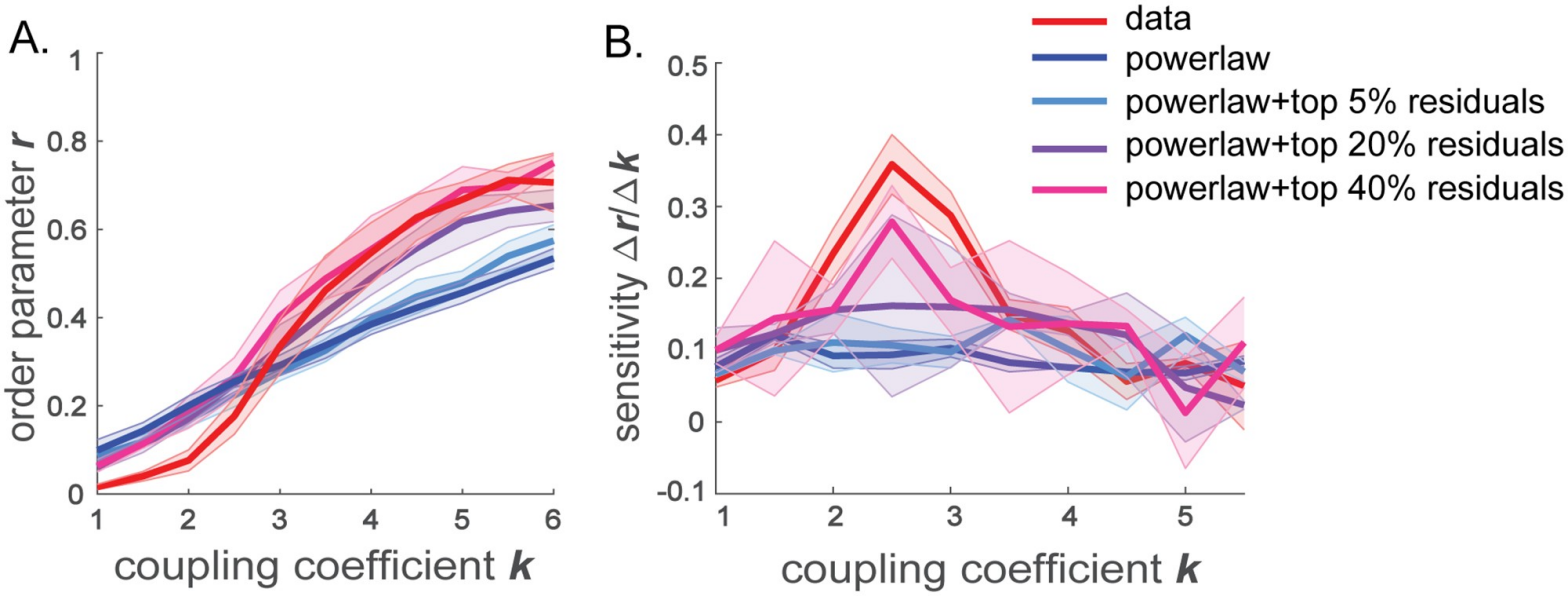
Order parameter *r* and its sensitivity for the power-law distance-dependent network with a fraction of the residual connections added. (A) Whole network order parameter *r* and (B) the sensitivity of the order parameter Δ*r*/Δ*k*, as a function of global coupling coefficient *k* for networks constructed by adding different percentiles of positive residual connections to the power-law approximated network.

We next examined what aspects of the residual connection strengths confer the network’s ability to span a wide range of coherence states. In previous studies on coupled oscillators, it has been found that even a small fraction of shortcuts in a small-world network significantly improves synchronization of the network [43, 53]. Motivated by this, we hypothesized that positive residual connections, namely, strong connections between distal brain regions, underlie the rapid transition in network synchronies. We tested this hypothesis by re-introducing small fractions of the positive residuals to the power-law distance-dependent network. As manifested in Fig 4A, adding just a small fraction (top 20 percentile) of the strongest positive residuals to the power-law generated network recovers the steep increase in order parameter with growing coupling coefficient (Fig 4, purple). As the fraction of positive residuals included in addition to the power-law network increases, the sensitivity of the order parameter as a function of the coupling coefficient resembles more of that of the real brain network (Fig 4B). This trend is also depicted by the maximum sensitivity measure which is at Γ_*k*_ = 0.1423 ± 0.0036, Γ_*k*_ = 0.1617 ± 0.1266, and Γ_*k*_ = 0.2785 ± 0.0506, respectively for top 5, 20, 40% of the positive residuals added to the power-law network, on the same edges as in the original data-driven whole-brain network.

Does the location of these strong connections have any significance in emergence of the rapid phase transition? To test whether the sensitivity of the network coherence to coupling coefficient can be recovered by simply adding the positive residuals anywhere to increase the overall connection strength of the power-law network, we studied the dynamics of the network constructed by relocating the positive residuals. We generated three networks with positive residuals relocated. In one of them, the positive residuals above the power-law relationship were positioned at random locations on the network (shuffled). In the other two, the positive residuals were preferentially relocated to the shortest 0.2% or to the longest 0.2% connections of the total edges. For the proximal-relocated network, the positive residual connections were added to connections between spatially close regions, by distributing the total positive residual connection strength among the connections between nodes within 570*μm*. For the distal-relocated network, the positive residuals were added to the connections between spatially distal regions, by distributing the total positive residual connection strength among the edges longer than 10500*μm*. The resulting networks thus maintain the total connection strengths of the real brain network, but have altered network structures.

**Fig 5:**
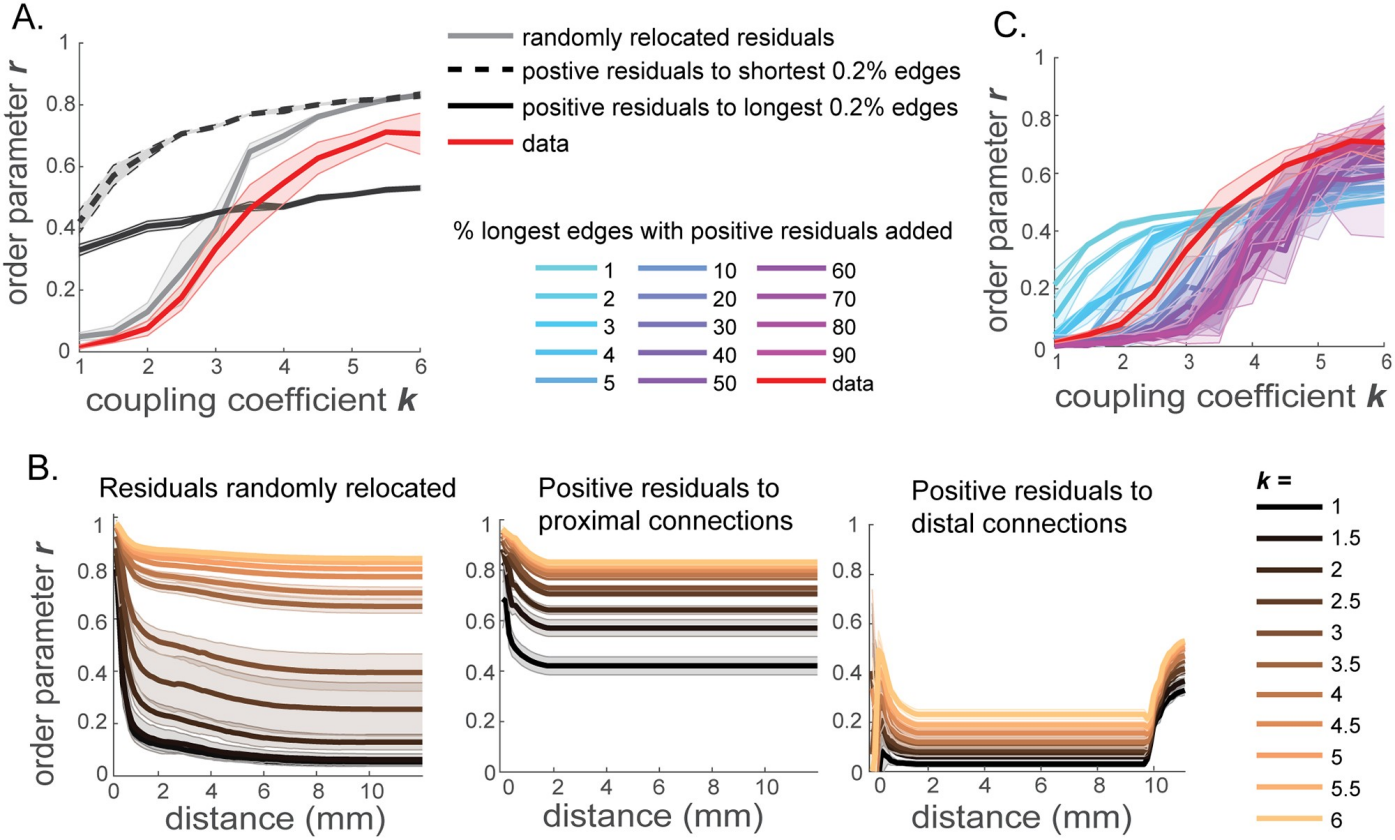
Synchronization measured when the network structure is altered but the total connection strength remains the same as the data-driven network. (A) Whole network order parameter *r* as a function of the global coupling coefficient *k*, for the networks generated by adding the residual connection weights to random locations (gray), by relocating positive residuals (averaged) to connections between spatially close regions (< 570*μm*) (black dotted), and by placing positive residuals on connections between distal regions (> 10500*μm*) (black solid). The order parameter for the data-driven brain network (red) is shown for comparison. (B) Order parameter as a function of the spatial scale of subnetworks, for the networks constructed by shuffling locations of residual connections (left, correspond to gray in panel A), by relocating positive residuals to nearby connections (middle, correspond to dotted black in panel A), and by relocating positive residuals to long-distance connections (right, correspond to black solid in panel A). (C) Order parameter *r* as a function of coupling coefficient *k* for networks constructed by adding the positive residual strengths to the longest 1-90% of edges (> 9404, 8830, 8470, 8175, 7946, 7168, 6265, 5626, 5063, 4521, 3992, 3455, 2867, 2147*μm*).

When the locations of the positive residuals are randomized and thus there are strong connection weights across multiple spatial scales, the dependence of network synchronization on *k* remains similar to that of the data-driven network, as portrayed by the order parameter in Fig 5A, in gray and Fig 5B, left. In other words, although the precise network structure is different from that of the data-driven network, the network with shuffled residuals maintains its sensitivity to the global coupling coefficient, rapidly changing network coherence states. However, the spatial structure of the order parameter is dependent on the precise locations of these positive residuals. In the network with positive residuals randomly relocated, there is a steeper decrease in order parameter with distance (Fig 5B, left), compared to the data-driven brain network (Fig 3B, left). This trend is depicted by the higher spatial coherence measure, Γ_*d*_ = 0.4091 ± 0.00017 for the network with randomized positive residuals, compared to the data-driven brain network (Γ_*d*_ = 0.1851 ± 0.0706).

When the positive residuals are relocated to proximal connections, the network coherence is no longer as sensitive to small changes in the global coupling coefficient as in the whole-brain network (Fig 5A, dotted black; B, middle), in spite of the unaltered total connection strengths. This trend is robustly maintained when the standard deviation in the natural frequency distribution is varied instead of the global coupling coefficient (Supporting Information S4 Fig). Similarly, when the positive residuals are moved to distal connections, the network coherence loses sensitivity as well (Fig 5A, solid black; B, right). Unlike the network with randomly relocated residuals, the networks with positive residuals relocated only to proximal or distal connections lack strong connections distributed across a range of spatial scales. Thus, connections that are stronger than predicted by the distance-dependence should be spread over varied lengths of edges, for the network to switch between localized and global coherence states with a small change in the global coupling coefficient.

In addition, we observe that when positive residuals are relocated to proximal connections, the overall order parameters across the spatial scales are higher (Fig 5B, middle), compared to the network constructed by placing positive residuals to distal connections (Fig 5B, right). This effect arises from the definition of the universal order parameter (Eqs 7 and 8), where each of the time-averaged phase difference 〈cos(*θ*_*i*_ − *θ*_*j*_)〉_*t*_ is weighted by the connection strength between the pair of oscillators *A*_*ij*_. When positive residuals are placed on proximal connections, the influence of the phase differences between nearby nodes, which increases the overall network order parameter, is emphasized more by larger connection strengths *A*_*ij*_. On the other hand, when the positive residuals are relocated to distal connections, although distal nodes are now more strongly coupled than before, the phase differences between distal nodes are still quite large. Therefore, in this case, the large phase differences between distal nodes which lower the overall order parameter, are strongly weighted by *A*_*ij*_, and thus, the overall network order parameters are maintained at low values. We also note that the order parameter rapidly increases at large distances in the power-law network with the residuals preferentially added to the longest edges (Fig 5B, right). This rapid increase stems from the relatively high values of the connections strengths of these longest edges (*A*_*ij*_) which induce large values of 〈cos(*θ*_*i*_ − *θ*_*j*_)〉_*t*_ between distal regions *i* and *j*. Therefore, the order parameters at the large spatial scales are increased by large values of *A*_*ij*_〈cos(*θ*_*i*_ − *θ*_*j*_)〉_*t*_ terms.

To further examine the relationship between the spatial spread of the strong connections and the sensitivity of synchronizability, we measured the order parameter in networks generated from the power-law approximation by placing the positive residual strengths to different fractions of the longest edges. In Fig 5C, we show the order parameter as a function of the coupling coefficient *k* when positive residuals are preferentially added back on edges that have lengths greater than various cutoff values. As the spatial scale over which the residuals are added widens, the sensitivity of the order parameter gradually increases. Notably, the sensitivity and the growth of the order parameter become comparable to those of the data-driven brain network when the percentile of the longest edges with added positive residuals reaches 5 − 10% of the total connections. This indicates that while it is important to have a spread of strong connections above the power-law prediction over multiple spatial scales, the spread does not have to extend all the way to the shortest edges of the network in order to generate high sensitivity of the order parameter observed in the data-driven brain network.

Our results show that the location of strong connections above the power-law dependence on distance is critical for generating a steep change in the order parameter. While the precise positions of the strong connections do not have to match those of the data-driven network to produce highly sensitive order parameter to the coupling coefficient, there should be a sufficient amount of strong connections across a range of spatial scales. Precise locations of the strong residuals, however, determine the order parameter’s dependence on the spatial scale, modulating spatial coherence patterns. In sum, the spatial structure of the network connectivity plays a key role in maintaining the brain’s ability to change its computational states with small perturbations, and such sensitivity cannot be achieved by simply matching the total network connection strengths. The structure does not have to precisely match that of the real brain network to maintain the high sensitivity. What is critical to maintain, rather, is some connections stronger than the simple distance-dependence distributed over the network. However, the precise connectivity structure is important for generating specific spatial coherence patterns in the network dynamics.

## Discussion

In this paper, we studied synchronization of a spatially constrained model of a weighted whole-brain network at the mesoscale, constructed from viral tracing experiments. The importance of linking connectivity structure and large-scale brain dynamics have been noted in previous studies [54–56]. In particular, the heterogeneity in structural connectivity has been proposed as a key underlying mechanism for certain brain network dynamic features such as functional hubs in resting state dynamics [56]. However, additional complexities in the anatomically precise, weighted and directed whole-brain network that are not captured by spatially-defined connectivity have been often overlooked. In this work, we propose possible computational roles of these additional complexities by studying their effects on network synchronizability. We found that the connectivity has a significant spatial dependence, with the connection strength decreasing with distance between the regions following a power law. However, by studying the network dynamics of phase oscillators, we found that a network generated by the simple spatial constraints alone cannot reproduce the full computational versatility of the mesoscopic whole-brain network. Rather, we need to consider additional complexities of the network structure to capture their possibly significant roles in neural computation. Specifically, we found that residual connections not explained by the power-law dependence on distance have a long positive tail, corresponding to strong connections between distal brain regions. By computing the recently proposed universal order parameter, we showed that these strong distal connections underlie sensitive dependence of network synchrony on perturbations in coupling coefficient (or intrinsic frequency distribution/noise), potentially responsible for the brain’s exceptional ability to change its computational states depending on stimulus and behavioral context. Furthermore, our analyses on networks constructed by adding a small fraction of strong positive residuals to the spatially-constrained connectivity, as well as networks with the positive residuals relocated to random, proximal, or distal connections, reveal the key element underlying the rapid switch between global and partial synchronies—strong connections distributed over varied spatial distances. In other words, the network’s sensitivity to perturbation cannot be reproduced by simply manipulating the overall connection strengths, as locations of positive residual connections should be taken into consideration. A spatially-constrained model plus an idiosyncratic sparse matrix which features strong connections between distal regions provides a parsimonious representation of the measured connectivity.

We hypothesize that the sharp transition in synchronization in the data-driven network, which is absent in the spatially-constrained power-law model, may underlie the brain’s ability to rapidly switch computational states [57]. Such a feature is known to be impaired in the brain under pathological conditions such as Alzheimer’s disease, suggested by studies showing more modular structures and decreased global efficiency in brain connectivity constructed from EEG, MEG, fMRI, and diffusion tensor tractography [58–61]. Moreover, there is an experimental evidence for disruption of long-range connections in Alzheimer brain network [60], in agreement with our model results. Therefore, the strictly distance-dependent power-law network which maintains localized synchronization across a range of coupling coefficients may explain aberrant network dynamics and computational impairments in Alzheimer brains. A more detailed future study on genetically-controlled mouse models of Alzheimer’s disease will shed light on the possible link between changes in structural connectivity and impairment in rapid phase transitions of the whole-brain network.

The increased sensitivity of the network synchronizability induced by strong long-range connections further implicates a tradeoff between cost-efficiency and high functional capacity in the brain network. Such tradeoff between wiring cost and computational capacity has been suggested as a network-generating principle in a number of previous studies [18, 62–67]. The power-law dependence of connection strengths on inter-regional distance reflects spatial and energetic constraints in the brain network. Indeed, if the brain connectivity is designed to exclusively optimize the wiring cost, we will observe strong connections only between proximal regions. Yet, we observe some idiosyncratic, strong long-range connections which are expensive in the mouse brain connectome. By showing that these strong distal connections may serve to promote rapid transitions between network synchronization states and possibly, computational states, our work points to a possible functional role afforded by the presence of the long-range connections despite their high metabolic costs.

In this paper, we infer the dynamics of the mesoscopic brain network by constructing a network of phase oscillators with the coupling strengths determined by the structural connectivity obtained by viral tracing experiments. Thus, while the structural connectivity is based on actual data, the dynamics we conferred on the network are arbitrary. Building a more realistic, data-driven dynamic network based on imaging experiments such as calcium-imaging, ECoG, LFP, and MEG will be a crucial future extension of our study of connecting the network structures to the network dynamics. Furthermore, for future studies, more biophysically-motivated neural mass models [68] would be necessary to capture realistic dynamics of the brain network that are not predicted by simple phase oscillator models. However, our simulations with phase oscillators, despite their generality, still make valuable predictions on computational roles of spatial structures of the mesoscopic whole-brain network, underlining the importance of spatially distributed, strong distal connections on the network dynamics.

## Methods

### Mouse whole-brain connectivity data

The mesoscopic mouse whole-brain connectivity was obtained from the Allen Mouse Brain Connectivity Atlas (http://connectivity.brain-map.org/), constructed based on anterograde viral tracing experiments in wild type C7BL/6 mice [10]. Based on the experimental data, a recently developed interpolative mapping algorithm was used to construct a model of whole brain connectivity at the 100 *μm*-voxel scale [22]. The voxel-based connection strengths were averaged over each brain region to produce a connectivity matrix with 244 brain regions per hemisphere as nodes, larger than the adjacency matrix of 213 pairs of nodes previously obtained from the linear model in [10]. For elements of the connectivity matrix, we use the *normalized projection density*, defined as the connection strength between two regions divided by the volume of the source and target regions. In order to account for the size of the source region, we also studied the relationship between the connection strength divided only by the size of the target region and the distance between two regions. In this case, however, the fit to either a power law or an exponential function was not very good which is not surprising given that the connection strengths that are not fully normalized with respect to the size of the source and the target is not an intrinsic quantity. For more details on the viral tracing experiments and the interpolative algorithm used to construct the connectivity matrix, see [10] and [22]. The connectivity matrix was first normalized to have values between 0 and 1. For the ipsilateral connection matrix, the diagonal entries were set to zeros removing self-connectivity, as done in [4, 20].

### Dependence of connection strengths on interregional distance

We fitted connection strengths as a function of interregional distance, where the distance between each pair of nodes was determined by computing the Euclidean distance in 3-dimensional coordinates between the centroids of the brain regions. Specifically, power-law functions for relationships between connection strength and interregional distance were fitted by using least squares on the log scale. For each of the ipsilateral and contralateral connectivity matrices, we found *α* and *β* by fitting the data to ${\tilde{A}}_{ij}=\alpha \cdot d_{ij}^{-\beta }+{∊}_{ij}$, where ${\tilde{A}}_{ij}$ denotes the connection strength from node *j* to node *i*, *d*_*ij*_ indicates the distance between nodes *i* and *j*, and *ϵ*_*ij*_ is the residual error. We obtained *α* = 6.92 × 10^6^ and *β* = 2.886 for ipsilateral connectivity, and *α* = 6.71 × 10^5^ and *β* = 2.685 for contralateral connectivity (Fig 1B). In agreement with previous studies on Allen Mouse Brain Connectivity data [18, 22], we found that the power law explains the relationship slightly better than the exponential dependence (ipsilateral r-square: 0.264 vs 0.257, rmse: 1.089 vs 1.095; contralateral r-square: 0.167 vs 0.135, rmse: 1.124 vs 1.146).

We also investigated the power-law constrained network where the relationship between connection strength and interregional distance was found on the real scale, using nonlinear least squares (Levenberg-Marquardt algorithm), which has a poorer explanatory power than linear least squares on the log-scale (r-square: 0.264 vs 0.157 (ipsilateral) / 0.167 vs 0.131 (contralateral)). While this method generated a different power-law function from the one found by least squares on the log-log scale, the dynamics on the power-law network obtained by using nonlinear least squares maintained the same core characteristics, distinct from the data-driven brain network– the order parameter is less sensitive to changes in the global coupling coefficient.

### Order parameter

In this section, we describe order parameters that were proposed previously [41, 42, 45, 46], demonstrating advantages of the recently developed universal order parameter [40] in our analysis.

In order to quantify network coherence in the original model of phase oscillators with all-to-all connectivity, Kuramoto introduced the complex order parameter [41, 42],

$$\begin{array}{c} \begin{array}{c} r\left(t\right)e^{\text{i}\psi (t)}\equiv \frac{1}{N}\sum_{i=1}^{N}e^{\text{i}\theta_{i}}, \end{array} \end{array}$$ (5)

where *ψ*(*t*) gives the average phase of all oscillators and *r*(*t*) describes the degree of phase coherence at time *t*. The overall phase coherence is measured by the absolute value of the complex order parameter averaged over time. We denote this value *r*_Kuramoto_, as the measure of the averaged phase differences of all pairs of oscillators:

$$\begin{array}{c} \begin{array}{cc} r_{\text{Kuramoto}}^{2} & \equiv \langle |r\left(t\right)e^{\text{i}\psi (t)}{|}^{2}\rangle_{t}\\ & =\langle \frac{1}{N^{2}}\sum_{i,j=1}^{N}e^{\text{i}(\theta_{i}-\theta_{j})}\rangle_{t}\\ & =\frac{1}{N^{2}}\sum_{i,j=1}^{N}\langle \cos\left(\theta_{i}-\theta_{j}\right)\rangle_{t}. \end{array} \end{array}$$ (6)

< … >_*t*_ denotes the average over time. However, this unweighted order parameter is not a good measure when comparing collective synchronizations in two networks described by different connectivity matrices, as it does not capture the topology of the networks.

To extend the use of order parameter to more general, weighted networks of oscillators, Restrepo et al [45, 46] proposed an order parameter which is defined as the average of local order parameters which measure the coherence of the inputs to each node. This parameter, however, does not capture partially phase-locked states well. Recently, Schroeder et al [40] proposed a new, “universal order parameter” to accurately measure phase coherence in weighted and directed networks of arbitrary topology, which overcomes the shortcomings of the previous order parameters. This newly proposed universal order parameter is defined as:

$$\begin{array}{c} \begin{array}{cc} r & \equiv \frac{1}{\sum_{i=1}^{N}k_{i}}\sum_{i,j=1}^{N}A_{ij}\langle Re(e^{\text{i}(\theta_{i}-\theta_{j})})\rangle_{t}\\ & =\frac{1}{\sum_{i=1}^{N}k_{i}}\sum_{i,j=1}^{N}A_{ij}\langle \cos\left(\theta_{i}-\theta_{j}\right)\rangle_{t} \end{array} \end{array}$$ (7)

where $k_{i}=\sum_{j=1}^{N}A_{ij}$ is the input strength of node *i*. Note that in unweighted binary networks, this measure represents in-degree [4]. This order parameter accounts for the network topology and its influence on the phase coherence, enabling a fair comparison between two topologically different weighted networks even when their total connection strengths are not matched. As this universal order parameter accurately captures partial synchrony within the network, different degrees of synchronization can be measured by order parameter of the whole network.

Furthermore, degree of coherence as a function of spatial extent can be obtained by computing the order parameter for subnetworks of different spatial scales. The order parameter *r* can be described as a function of distance *d*:

$$\begin{array}{c} \begin{array}{cc} r(d) & \equiv \frac{1}{\sum_{i=1}^{N}k_{i}}\sum_{i=1}^{N}\sum_{j\in \gamma (i,d)}A_{ij}\langle Re(e^{\text{i}(\theta_{i}-\theta_{j})})\rangle_{t}\\ & =\frac{1}{\sum_{i=1}^{N}k_{i}}\sum_{i=1}^{N}\sum_{j\in \gamma (i,d)}A_{ij}\langle \cos\left(\theta_{i}-\theta_{j}\right)\rangle_{t} \end{array} \end{array}$$ (8)

where *γ*(*i*, *d*) indicates the set of nodes within spatial distance *d* from node *i*. *k*_*i*_ = ∑_*j*∈*γ*(*i*,*d*)_ *A*_*ij*_ is the total connection strength of node *i* when the subnetwork composed of nodes within distance *d* from node *i* is considered. The order parameter of the whole network is obtained when *d* = size of the network (11752*μm* for ipsilateral and 11955*μm* for contralateral connectivity).

All of the MATLAB code used to numerically compute time-series data of coupled oscillators and the order parameters on the mouse whole-brain network from [10, 22] and the power-law approximated network are available at https://github.com/AllenInstitute/Choi2019_ConnectomeSynchrony.

## Supporting information

S1 Fig Network synchronizability in the single hemisphere network with ipsilateral connections only. Universal order parameter over a range of global coupling coefficient *k*, for the data-driven mouse brain network (red) and the power-law estimated network (blue) of a single hemisphere with ipsilateral connectivity.
(TIF)

S2 Fig Network synchronizability with varied *σ*_*n*_ and *σ*_*d*_. The order parameters for the data-driven mouse brain network (red) and the power-law constrained network (blue) are plotted as a function of either (A) the dispersion in the intrinsic frequency distribution (*σ*_*d*_) or (B) the standard deviation of the additive white noise (*σ*_*n*_). The white noise is fixed at 0 (*σ*_*n*_ = 0) while the frequency dispersion (*σ*_*d*_) is varied. Homogeneous frequencies across the network are assumed (*σ*_*d*_ = 0) while the amount of the white noise (*σ*_*n*_) is varied. (C) The order parameters for the data-driven network (red) and the power-law constrained network (blue) as a function of the coupling coefficient *k* as in Fig 3(C) in the main text, with heterogeneous intrinsic frequencies across the network (*σ*_*d*_ = 0.2) and an additive white noise (*σ*_*n*_ = 2). The order parameters are averaged over 100 repeats of simulations.
(TIF)

S3 Fig Network synchronizability with intrinsic frequency values in theta- and beta-frequency ranges. The order parameters for the data-driven mouse brain network (red) and the power-law constrained network (blue) are plotted for varied coupling coefficient *k*, when the intrinsic frequencies of the networks are in the frequency range of either theta-oscillations (*ω*_*i*_ = 8(*Hz*) for all *i*; left) or beta-oscillations (*ω*_*i*_ = 20(*Hz*) for all *i*; right).
(TIF)

S4 Fig Synchronization when the network structure is altered, with the dispersion in natural frequncies varied. The same simulations as with Fig 5 in the main text, but *σ*_*d*_ is varied while *σ*_*n*_ = 0 and *k* = 2, with no time delays. (A) The order parameters are shown for the network with the residual connections randomly relocated (gray), the network with the positive residuals placed on shortest-edges (< 500*μm*, green), and the data-driven brain network (red) (B) Order parameter as a function of distance, for the network with randomly placed residuals (left) and the network with positive residuals on poximal connection (right).
(TIF)
