# Supplementary figures and images for: Synchronization dependent on spatial structures of a mesoscopic whole-brain network
Source: PLoS Comput Biol. 2019 Apr 23;15(4):e1006978. doi: 10.1371/journal.pcbi.1006978 (PMC6499430; doi:10.1371/journal.pcbi.1006978)

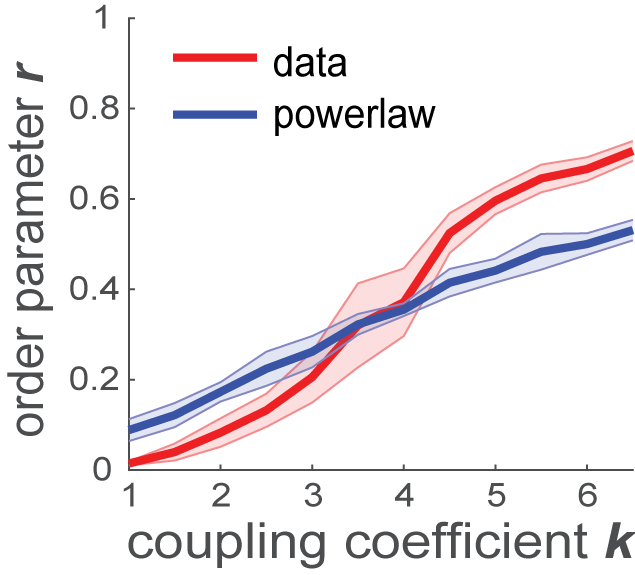

Supplement: S1 Fig — Universal order parameter over a range of global coupling coefficient k, for the data-driven mouse brain network (red) and the power-law estimated network (blue) of a single hemisphere with ipsilateral connectivity. (TIF) [file pcbi.1006978.s001.tif]

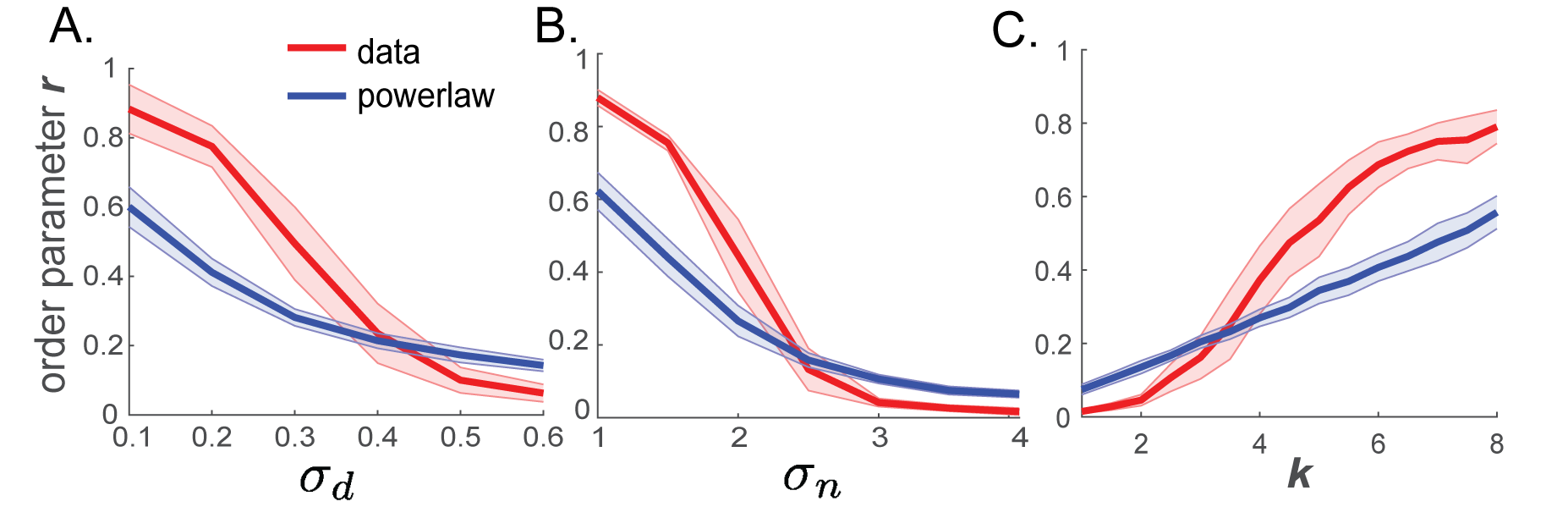

Supplement: S2 Fig — The order parameters for the data-driven mouse brain network (red) and the power-law constrained network (blue) are plotted as a function of either (A) the dispersion in the intrinsic frequency distribution (σd) or (B) the standard deviation of the additive white noise (σn). The white noise is fixed at 0 (σn = 0) while the frequency dispersion (σd) is varied. Homogeneous frequencies across the network are assumed (σd = 0) while the amount of the white noise (σn) is varied. (C) The order parameters for the data-driven network (red) and the power-law constrained network (blue) as a function of the coupling coefficient k as in Fig 3(C) in the main text, with heterogeneous intrinsic frequencies across the network (σd = 0.2) and an additive white noise (σn = 2). The order parameters are averaged over 100 repeats of simulations. (TIF) [file pcbi.1006978.s002.tif]

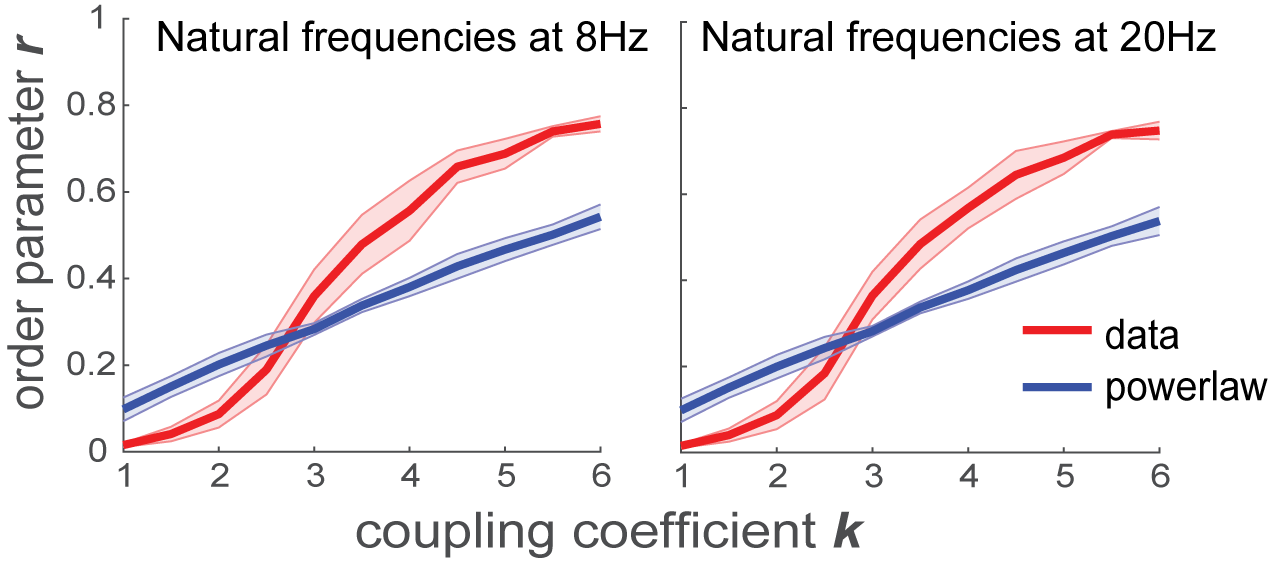

Supplement: S3 Fig — The order parameters for the data-driven mouse brain network (red) and the power-law constrained network (blue) are plotted for varied coupling coefficient k, when the intrinsic frequencies of the networks are in the frequency range of either theta-oscillations (ωi = 8(Hz) for all i; left) or beta-oscillations (ωi = 20(Hz) for all i; right). (TIF) [file pcbi.1006978.s003.tif]

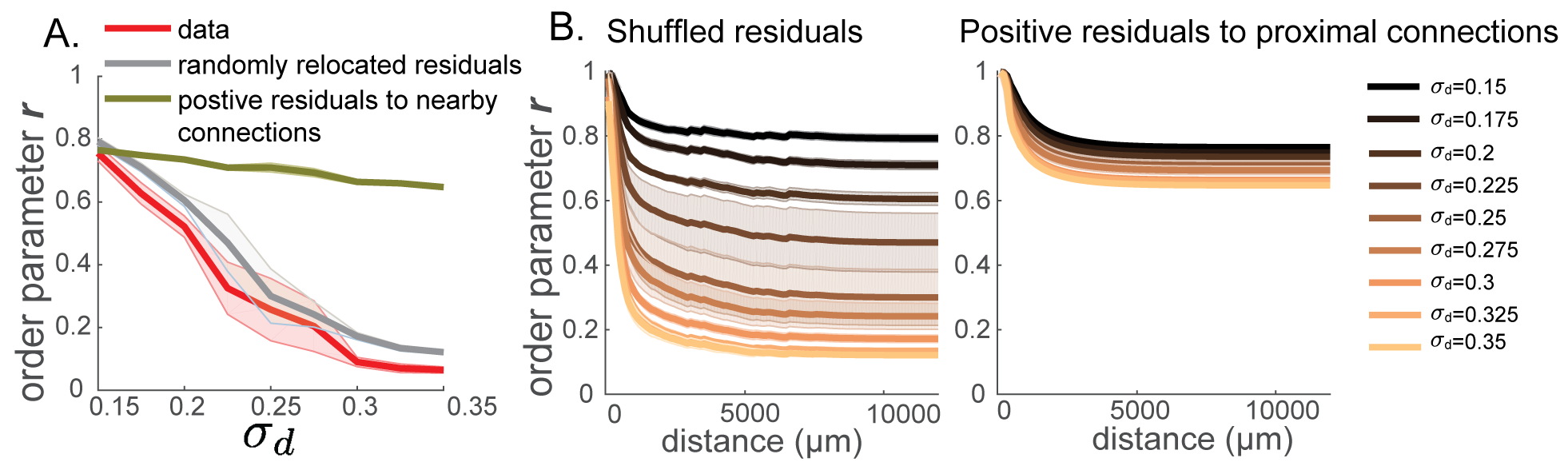

Supplement: S4 Fig — The same simulations as with Fig 5 in the main text, but σd is varied while σn = 0 and k = 2, with no time delays. (A) The order parameters are shown for the network with the residual connections randomly relocated (gray), the network with the positive residuals placed on shortest-edges (< 500μm, green), and the data-driven brain network (red) (B) Order parameter as a function of distance, for the network with randomly placed residuals (left) and the network with positive residuals on poximal connection (right). (TIF) [file pcbi.1006978.s004.tif]
